# Could a Multi-Marker and Machine Learning Approach Help Stratify Patients with Heart Failure?

**DOI:** 10.3390/medicina57100996

**Published:** 2021-09-22

**Authors:** Manuela Lotierzo, Romain Bruno, Amanda Finan-Marchi, Fabien Huet, Eran Kalmanovich, Glaucy Rodrigues, Anne-Marie Dupuy, Jérôme Adda, David Piquemal, Sylvain Richard, Jean-Paul Cristol, François Roubille

**Affiliations:** 1 PhyMedExp, Université de Montpellier, INSERM, CNRS, 34295 Montpellier, France; m-lotierzo@chu-montpellier.fr (M.L.); amandafinan@yahoo.com (A.F.-M.); glaucyrodrigues@gmail.com (G.R.); jp-cristol@chu-montpellier.fr (J.-P.C.); 2 CHU de Montpellier, Département de Biochimie et Hormonologie, Université de Montpellier, 34090 Montpellier, France; am-dupuy@chu-montpellier.fr; 3 ACOBIOM, 1682 Rue de la Valsière, Cap Delta, 34790 Montpellier, France; bruno@acobiom.com (R.B.); piquemal@acobiom.com (D.P.); 4 CHU de Montpellier, Département de Cardiologie, Université de Montpellier, 34090 Montpellier, France; f-huet@chu-montpellier.fr (F.H.); kalmanovicheran@gmail.com (E.K.); adda.jerome@gmail.com (J.A.)

**Keywords:** Machine Learning strategy, HFpEF, blood signature, HF patient stratification, multimarker approach

## Abstract

Half of the patients with heart failure (HF) have preserved ejection fraction (HFpEF). To date, there are no specific markers to distinguish this subgroup. The main objective of this work was to stratify HF patients using current biochemical markers coupled with clinical data. The cohort study included HFpEF (*n* = 24) and heart failure with reduced ejection fraction (HFrEF) (*n* = 34) patients as usually considered in clinical practice based on cardiac imaging (EF ≥ 50% for HFpEF; EF < 50% for HFrEF). Routine blood tests consisted of measuring biomarkers of renal and heart functions, inflammation, and iron metabolism. A multi-test approach and analysis of peripheral blood samples aimed to establish a computerized Machine Learning strategy to provide a blood signature to distinguish HFpEF and HFrEF. Based on logistic regression, demographic characteristics and clinical biomarkers showed no statistical significance to differentiate the HFpEF and HFrEF patient subgroups. Hence a multivariate factorial discriminant analysis, performed blindly using the data set, allowed us to stratify the two HF groups. Consequently, a Machine Learning (ML) strategy was developed using the same variables in a genetic algorithm approach. ML provided very encouraging explorative results when considering the small size of the samples applied. The accuracy and the sensitivity were high for both validation and test groups (69% and 100%, 64% and 75%, respectively). Sensitivity was 100% for the validation and 75% for the test group, whereas specificity was 44% and 55% for the validation and test groups because of the small number of samples. Lastly, the precision was acceptable, with 58% in the validation and 60% in the test group. Combining biochemical and clinical markers is an excellent entry to develop a computer classification tool to diagnose HFpEF. This translational approach is a springboard for improving new personalized treatment methods and identifying “high-yield” populations for clinical trials.

## 1. Introduction

Heart failure (HF) represents a public health problem with significant medical, societal, and economic impacts (repeated hospitalizations). Half of the HF patients have a preserved ejection fraction but an impaired diastolic function (HFpEF). This subtype of HF is multifactorial and complex, with different comorbidities, gender, and aging issues [1,2,3]. Currently, diagnosis is based on cardiac imaging, echocardiography, or catheterization. Natriuretic peptides (NT-pro-BNP or BNP) are presently considered for diagnosis and monitoring. Although there is no reliable and specific biomarker to identify HFpEF, soluble suppression of tumorigenicity 2 (sST2), a biomarker of fibrosis and inflammation, and circulating cells represent emerging biomarkers for HFpEF [4,5,6,7,8,9]. Early identification of HFpEF before its onset is highly desirable. In this work, we designed a multi-biomarkers strategy associated with a cutting-edge Machine Learning (ML) approach to distinguish between HFpEF and HFrEF populations.

## 2. Materials and Methods

The study population included HFpEF (*n* = 24) and HFrEF (*n* = 34) patients. Inclusion criteria were age (>65 years), established HF, electrocardiogram (ECG), echocardiography, previous hospitalizations for HF, and follow-up. The local Ethics Committee approved the study of Montpellier University Hospital (N° DC-2016-2882). All enrolled patients provided their informed, signed consents. Criteria for non-inclusion were hemodynamic instability (cardiogenic shock) and any condition leading to a prognosis of fewer than seven days. We classified patients on a clinical basis with cardiac imaging (LVEF > 50% for HFpEF; LVEF < 50% for HFrEF). Importantly, HFpEF is diagnosed, following the guidelines, and diagnostic criterai were consistent with the recent criteria presented by the European guidelines on HF: the diagnosis is based on (1) symptoms and signs of HF, (2) with evidence of structural and/or functional cardiac abnormalities (such as left ventricular diastolic dysfunction/raised Left ventricular filling pressures) and/or (3) raised natriuretic peptides (NPs), and with an LVEF ≥ 50%. We collected clinical and biological data for each group. Routine blood tests consisted of measuring creatinine, urea, and estimated glomerular filtration rate (eGFR), N-terminal pro-B-type natriuretic peptide (NT-proBNP) and high-sensitivity cardiac troponin T (hs-cTnT), inflammatory marker C-reactive protein (CRP), and iron metabolism (transferrin—saturation coefficient—TSC%—and serum ferritin). Additionally, we collected an EDTA blood sample to measure sST2 in plasma. First, we performed a logistic regression analysis between the two groups of patients. Logistic regression analysis is commonly used to establish the significance of certain parameters in diagnosis or outcome prediction. Then, we carried out a multivariate factorial discriminant analysis (FDA) blindly on all recruited patients ignoring the established clinical data-based groups. These analyses were assisted by a Monte-Carlo permutation test for assessing statistical significance in the discrimination analysis. Finally, a novel approach based on ML was applied to optimize the variables selection and then compute a solution to provide a blood signature to distinguish HFpEF and HFrEF. All predictors previously mentioned were included in the ML prediction model, notably sex, age, and biochemical parameters (urea, creatinine, eGFR, NT-proBNP, hsTnT, CRP, sST2, ferritin, and %TSC). We applied a hold-out strategy for this approach, constituting a training set of 21 samples and validation of 16 samples, and then finally tested on 17 samples [10]. Each group was sequentially randomized, and then the algorithm evaluated the key statistical parameters. This process was performed 10,000 times (see more on methods in Supplementary Data).

## 3. Results

### 3.1. Logistic Regression Analysis

Although linear relation for each predictor was investigated to yield the best performance prediction score, demographic characteristics and clinical biomarkers showed no statistical significance to differentiate HFpEF and HFrEF patient subgroups based on logistic regression analysis (Type 1 error alpha set at 5%) (Table 1).

Nevertheless, it is essential to note that, as expected, the HFpEF group was characterized by older age (74 versus 69 years old) and that the NTproBNP value was lower for the target group. In addition, the inflammation state proved by the CRP, and sST2 values was more critical for the HFpEF group. We measured ferritin and TSC in both groups of patients to explore iron status. A ferritin level lower than 100 µg/L or lower than 100–300 µg/L together with a transferrin saturation coefficient (TSC) lower than 20% was considered as iron deficiency (ID) [11]. Furthermore, ID seemed prevalent in the HFpEF population compared to the HFrEF population, with more deficient functional capacity and quality of life in these patients [12,13]. Although the difference was not significant, our results showed lower ferritin values for the HFpEF group (138 µg/L versus 154 µg/L) with TSC% less than 20 in this group of patients (17% versus 25%).

### 3.2. Multivariate Factorial Discriminant Analysis

**Figure 1:**
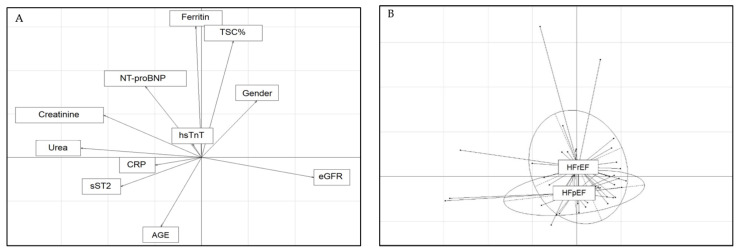
Arrow (**A**) and class (**B**) plots for clinical and biochemical metrics. (**A**) Arrow plot for clinical and biochemical metrics as a projection on two axes of the whole metrics used in the prediction model. (**B**) Class plot showed the same axis set-up but with the projection of the two respective HF groups of patients.

A discriminant analysis was implemented to determine a potential correlation between the metrics. We evaluated the multivariate factorial discriminant analysis (FDA) model using a Monte Carlo permutation test to assess the statistical significance of the discriminant analysis. We set the Type 1 error threshold at 5%. The Monte Carlo test yielded a *p*-value of 0.15. Although not statistically significant, the FDA provided a stratification of the two HF groups considering the parameters of the entire cohort of HF patients apart from the LVEF. As shown in Figure 1, this blinded approach resulted in a stratification of patients similar to that established by LVEF analysis.

The most discriminating anti-correlation vertical arrows corresponded to age and iron metabolism (ferritin + TSC%). As expected, the horizontal axes showed the perfect anti-correlation between eGFR on one side and creatinine and urea values on the other side. To a lesser extent, the age arrow was also anti-correlated to sex and NT-proBNP. While the hsTnT value did not seem to discriminate between the two subgroups, NT-proBNP was inversely correlated with sST2, confirming a potential involvement in two different pathophysiological states. Finally, CRP correlated with sST2, highlighting the link between sST2 and the inflammation process (Figure 1A). The class plot showed the same axis set-up and the projection of the two respective HF groups of patients (Figure 1B). The arrow plots impacted the labeled gravity center for each group. The two groups were slightly segregated around the vertical axis. Thus, the HFrEF group was dominated by higher values of ferritin and TSC% than the HFpEF group. Therefore, the verticality of the HFrEF group gravity center was mainly determined by age, lower than the HFpEF group, and sex with a predominance of men.

### 3.3. Machine Learning

We developed a Machine Learning (ML) strategy using the same variables in a genetic algorithm approach. We present the results from the model in Table 2. A hold-out method was applied consisting of a training set of 21 samples and the validation of 16 samples, then finally tested on 17 pieces. With standard clinical and biochemical variables, ML provided results with an accuracy (ratio of the number of correct predictions to the total number of input samples) of 69% and 64%, respectively, for the validation and test groups. Sensitivity, defined as the percentage of actual positives correctly identified, was 100% for the validation and 75% for the test group. Concerning specificity, notably measuring the rate of true negatives correctly identified, the percentage values were 44% and 55% for the validation and test groups, respectively, which we partially expected because of the small number of samples. Lastly, the precision or the positive predictive value was acceptable, with 58% in the validation and 60% in the test group.

## 4. Discussion

There are pathogenetic differences among HF subtypes associated with different risk factors supporting the need for better discrimination among patient subgroups [14]. The diagnosis of HFpEF remains a significant challenge, especially at an early stage of the disease. There are no precise biomarkers for it, and therapies are not specifically suitable. In addition, HFpEF is highly heterogeneous, making it difficult to reach a consensus on which predictors to use reliably [15]. The critical need for better stratification was underlined after the uncertain results of the PARAGON trial [2,3]. The angiotensin receptor-neprilysin inhibitor sacubitril–valsartan, currently approved to treat patients with HFrEF, missed statistical significance for the primary outcome (hospitalization or death from a cardiovascular origin time-to-event analysis) in HFpEF patients. Nevertheless, this trial suggested a potential benefit for patients with ‘mid-range LVEF and women, pointing out the potential specific relevance of sacubitril–valsartan in HFpEF patients [1,3]. This trend was consistent with network analysis showing that the biomarkers profiles for HFpEF and HFrEF are different [16,17].

A novel typology of markers based on ML strategy combining other parameters for discrimination of patient subgroups seemed achievable for HF. In this work, biochemical markers coupled to demographic data of an HF cohort (11 predictors) were introduced randomly into a computer analysis without knowledge of the patients’ LVEF. We attempted to generate an algorithm capable of disentangling confidential information based on a clustering model between specific sub-populations despite the small sample size. While a standard multivariate analysis showed non-statistical significance (*p* = 0.15), our approach enabled the identification of the two groups of patients. In the ML approach, although the small cohort investigated did not allow to conclude definitively, the results doubtless showed a promising way to discriminate between two populations of HF patients, and the association of biochemical and demographic variables was proved to be an excellent entry to build a classification tool to diagnose HFpEF. Nevertheless, one limitation of this work is the availability of relevant substrates to derive ML algorithms. Modern Machine Learning algorithms are robust and highly predictive when they rely on large data sets. On the other hand, the type of approach on small populations used in this work is emerging as reliable and safe, although more challenging. The methodology we used performed transfer learning, an up-and-coming technique consisting in transferring the knowledge learned in one dataset and apply it to another dataset. Flexibility is much more important than classic machine-learning methodology, using this kind of adaption of deep neural networks. By their very nature, ML tools will only be as robust as the data they see [18]. The specificity of this type of approach must be improved since we achieved only 44% and 55% for the validation and test groups, respectively. Thus, this prediction model does not yet allow the net discrimination between the two HF subpopulations. One axis of progress rates is developing the present research to a larger cohort and integrating a set of genomic variables to help measure the robustness of predictions made and set a realistic benchmark for predictive early diagnostic [15,19]. Therefore, the reasonable idea is to generalize this methodology to validate our pilot study using a larger cohort or selecting different parameters. This approach followed previous studies in various fields, including the cardiovascular domain, to prospectively evaluate predictive ML algorithms in a real-world setting [18,19,20]. In a study focusing on a cohort of 149 patients from the Framingham Heart Study, the comparison of five ML models with one conventional logistic regression model to predict HFpEF risk and to identify subgroups based on gene expression data showed that the kernel partial least squares with the genetic algorithm model exhibited the best performance in predicting patient risk of death [15]. This discriminatory capacity approach can potentially be applied to more complex problems, particularly recognition within the same HFpEF population for better stratification and more precise patient management [21]. The challenge of developing ML methods for small data sets combining different biological markers applied to small cohorts warrants further investigation. New data from cellular and omic biomarkers can be easily incremented to enrich the profile and identify the same pathology subtypes. Efforts to understand HF could also push the boundaries of clinical practice beyond the simple dichotomy between HFrEF and HFpEF.

## Figures and Tables

**Table 1:** Demographic characteristics, clinical biomarkers values, and results from logistic regression.

	Study Population, *n* = 58	Control Group, *n* = 34, HFrEF	Study Group, *n* = 24, HFpEF	*p* Value
	Median (1°–3° Quartile),			
Age, years	All= 68 (59–79) F = 75 (67–82) M = 67 (55–78)	69 (59–78)	74 (60–82)	
Gender, *n* (%)	F = 18 (32) M = 40 (68)	7 (21) 27 (79)	11 (50) 13 (50)	0.321
LVEF, %	40 (30–50)	30 (23–37)	60 (50–60)	NA
Urea, mmol/L	8.3 (6.55–12)	8.75 (6.75–12)	7.6 (6–10)	0.553
Creatinine, µmol/L	101(87–130)	105 (95–139)	87 (75–104)	0.663
eGFR, mL/min–1,73 m^2^	61 (45–78)	56 (41–77)	65 (47–81)	0.779
NT-proBNP, pg/mL	1457 (465–3675)	1546 (491–4092)	1090 (460–2803)	0.237
hs-cTnT	33 (16–48)	33 (27–48)	27 (11–45)	0.488
CRP, mg/L	2.6 (1–6)	2.4 (1–4.3)	5 (1–8.6)	0.485
sST2, ng/mL	33.4 (24–56)	32.0 (22–52)	44 (25–64)	0.256
Ferritin, µg/L	146 (87–296)	154 (122–338)	138 (60–183)	0.526
TSC, %	21.0 (16–28)	25.0 (18–29)	17 (13–24)	0.232

HFrEF: heart failure with reduced ejection fraction; HFpEF: heart failure with preserved ejection fraction; F: female, M: male; LVEF: left ventricular ejection fraction; eGFR: estimated glomerular filtration rate; NT-proBNP: N-terminal pro–B-type natriuretic peptide; hs-cTnT: high-sensitive cardiac troponin T; CRP: C—reactive protein; sST2: soluble suppression of tumorigenesis-2; TSC: transferrin saturation coefficient.

**Table 2:** Computing model results for discriminating HFpEF versus HFrEF.

	Samples	Accuracy, %	Sensitivity, %	Specificity, %	Precision, %
Validation	16	69	100	44	58
Test	17	64	75	55	60

## Supplementary Materials

The following are available online at https://www.mdpi.com/article/10.3390/medicina57100996/s1, Supplementary Data.
