# Supplementary material for: Could a Multi-Marker and Machine Learning Approach Help Stratify Patients with Heart Failure?
Source: Medicina (Kaunas). 2021 Sep 22;57(10):996. doi: 10.3390/medicina57100996 (PMC8538712; doi:10.3390/medicina57100996)
Supplement: Supplementary file 1 [file medicina-57-00996-s001.zip › medicina-1322927-supplementary.pdf]

Supplementary (Method)

We used a “hold-out” strategy where we randomly sampled 40% of the data for the training set, 30% of the data for the test set and 30% of the data for the validation set. We performed this process 10.000 times and then used an adaptation of genetic algorithm (mydatamodels.com web app). This methodology uses a combination of symbolic regression (a type of regression analysis that searches the space of mathematical expressions to find the model that best fits a given dataset) and evolutionary programming (a stochastic optimization strategy similar to genetic algorithms, but instead places emphasis on the behavioral linkage between parents and their offspring, rather than seeking to emulate specific genetic operators as observed in nature). Evolutionary programming is similar to evolution strategies, although the two approaches developed independently. This allowed to further diminish the number of pertinent variables and push a more robust predictive model.

John R. Koza; Martin A. Keane; James P. Rice (1993). "Performance improvement of machine learning via automatic discovery of facilitating functions as applied to a problem of symbolic system identification"

L.J. Fogel and D.B. Fogel (1986) “Artificial intelligence through evolutionary programming,” Final Report, Contract PO-9-X56-1102C-1, U.S. Army Research Institute.
